# A Model-Based Spike Sorting Algorithm for Removing Correlation Artifacts in Multi-Neuron Recordings

**DOI:** 10.1371/journal.pone.0062123

**Published:** 2013-05-03

**Authors:** Jonathan W. Pillow, Jonathon Shlens, E. J. Chichilnisky, Eero P. Simoncelli

**Affiliations:** 1 Center for Perceptual Systems, Department of Psychology and Section of Neurobiology, The University of Texas at Austin, Austin, Texas, United States of America; 2 The Salk Institute, La Jolla, California, United States of America; 3 Howard Hughes Medical Institute and Center for Neural Science and Courant Institute, NYU, New York, New York, United States of America; Rutgers University, United States of America

## Abstract

We examine the problem of estimating the spike trains of multiple neurons from voltage traces recorded on one or more extracellular electrodes. Traditional spike-sorting methods rely on thresholding or clustering of recorded signals to identify spikes. While these methods can detect a large fraction of the spikes from a recording, they generally fail to identify synchronous or near-synchronous spikes: cases in which multiple spikes overlap. Here we investigate the geometry of failures in traditional sorting algorithms, and document the prevalence of such errors in multi-electrode recordings from primate retina. We then develop a method for multi-neuron spike sorting using a model that explicitly accounts for the superposition of spike waveforms. We model the recorded voltage traces as a linear combination of spike waveforms plus a stochastic background component of correlated Gaussian noise. Combining this measurement model with a Bernoulli prior over binary spike trains yields a posterior distribution for spikes given the recorded data. We introduce a greedy algorithm to maximize this posterior that we call “binary pursuit”. The algorithm allows modest variability in spike waveforms and recovers spike times with higher precision than the voltage sampling rate. This method substantially corrects cross-correlation artifacts that arise with conventional methods, and substantially outperforms clustering methods on both real and simulated data. Finally, we develop diagnostic tools that can be used to assess errors in spike sorting in the absence of ground truth.

## Introduction

Action potentials, often referred to as “spikes”, are the fundamental unit of communication in much of the nervous system. The problem of estimating the timing and identity of spikes from extracellular analog voltage recordings, generally known as *spike sorting*, was originally studied for recordings of single neurons on single electrodes. However, many newly developed multi-electrode recording techniques aim to examine the simultaneous activity of populations of neurons in a neural circuit [1]–[6]. With a few notable exceptions, spike-sorting methodologies have not kept up.

Most spike-sorting techniques rely on the observation that individual neurons produce stereotyped spike waveforms. The earliest methods, developed for single neurons recorded on single electrodes, rely on the basic strategy of *matched filtering*: the electrode waveform is compared against a temporally sliding template and a spike is identified whenever the two are found to match within some tolerance. This methodology predates the era of digital computers, when the matching was done using hand-adjusted threshold triggers on an oscilloscope [7]. A form of this technique is still widely used in single-cell electrophysiology, where the electrode position is adjusted to maximize the waveform amplitude of one cell. In general, matched filtering is known to be optimal for detecting isolated waveforms of known shape and amplitude in a background of white noise [8]. However, this optimality degrades quickly when waveforms of more than one spike overlap, as is common in extracellular recordings. In fact, much of the “background” noise in neural recordings is likely due to spikes of other cells [9]; if those spikes are large enough, any methodology based on template matching is likely to fail [10], [11]. Moreover, because it typically requires hand-adjustment of thresholding parameters, matched filtering is not practical for sorting multi-electrode data from large electrode arrays.

Modern methods have extended the matched filtering strategy to identify spikes from multiple cells, measured with multiple electrodes, by first selecting short segments of the recorded waveforms during which the voltage exceeds some threshold, and then identifying individual neurons and their spikes by identifying clusters within the space spanned by these segments [12], [13]. A variety of different *clustering* methodologies have been explored as well as new methods for selecting appropriate waveform features (e.g., [14]–[19]). But clustering methods, just like the matched filtering methods that preceded them, exhibit failures when spikes from two or more cells are superimposed [4], [20]–[22]. Despite these drawbacks, clustering methods are the current *de facto* standard; they are distributed in analysis software by manufacturers of multi-electrodes [23] and are considered adequate for most experiments in which a relatively small number of neurons are recorded or analyses in which a small fraction of errors are acceptable.

We suggest that the errors that occur when spikes are superimposed are more severe than is commonly assumed. First, these errors are not random, but highly systematic, and can complicate conclusions regarding the occurrence of near-synchronous spikes, and their role in network activity. Accumulating evidence suggests that correlated or synchronized firing amongst cells within a network is likely to be far more prevalent than previously believed. For example, recent analysis of retinal ganglion cells show that synchronous spikes constitute up to 60% of all spiking activity and can occur in events constituting a large fraction of the neurons recorded [2], [24]. Second, as recording technology advances, increases in both the number of electrodes and the recording fidelity of electrodes lead to ever more frequent occurrences of spike superposition. Thus, spike sorting solutions that directly address the superposition problem are clearly needed.

Several recent papers have addressed the problem of spike sorting while explicitly addressing the problem of overlapping spikes [4], [25]–[28]. (See Discussion for a more detailed comparison). Here we make several new contributions to the study of this problem. First, we carefully examine the failure of clustering methodologies in cases where spikes from multiple neurons overlap. We examine how these failures lead to systematic artifacts which can be used to diagnose any spike-sorting algorithm in the absence of ground-truth. Second, we propose a framework for spike sorting based on a simple generative model of extracellularly recorded data. This model formalizes a set of prior beliefs and assumptions about neural spike trains and waveforms and how these signals combine to generate a noisy voltage waveform. In particular, this model specifies that overlapping spikes from nearby neurons superimpose linearly in the recorded voltage signal. We introduce a greedy algorithm – “binary pursuit” – for obtaining the approximate *maximum a posteriori* (MAP) estimate of the spike trains given the voltage data under this model. We demonstrate that in comparison to clustering methods, binary pursuit can reduce both the number of missed spikes and the rate of false positives. Finally, we develop a new method for assessing the spike sorting error rate in the absence of ground truth, and we use this to demonstrate the quality of our results on real data.

## Results

### Failures of Clustering Methods

We begin by examining the geometry of extracellular spike recordings in order to provide an intuitive illustration of the limitations of clustering methods, and to motivate our proposed methodology. Clustering methods for spike sorting follow several generic steps. First, putative spike times and their associated waveforms are isolated from an analog voltage trace. Then, the voltage traces in the vicinity of these spike times are grouped into clusters. The centroid of each cluster is identified as the spike waveform of a neuron, and all traces that fall within a cluster are then labelled as spikes of the corresponding neuron (see Methods). Although the details vary, these steps constitute the primary elements of most spike sorting algorithms described in the literature [13], [16], [21] as well as most commercially available spike sorting systems [23].

**Figure 1:**
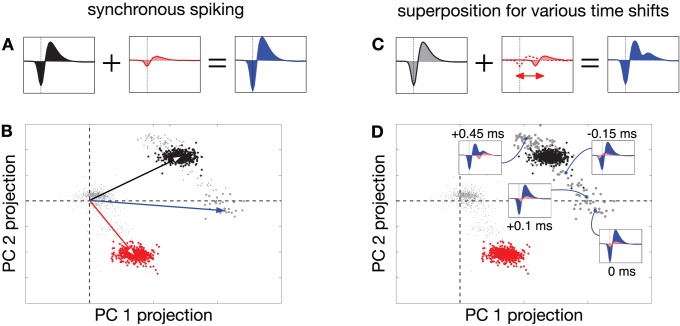
Geometric picture of failures in clustering-based spike sorting, with multi-electrode retinal data [29]. (**A**) Synchronous spike waveforms on a single extracellular electrode from two different neurons (black and red), which sum linearly to form a new waveform (blue) when these neurons fire synchronously. (**B**) Spike waveforms from these same two neurons projected into a two-dimensional linear feature space. Each point in this space corresponds to a single recorded waveform. Black and red vectors indicate the waveforms shown in (A), and the corresponding clusters of colored points around each vector indicate the samples that were assigned to each neuron. Synchronous spikes from these two neurons give rise to voltage waveforms that lie near the sum of these two vectors (blue vector), and these points (gray) are generally discarded as outliers. (**C–D**) More generally, overlapping spikes with different temporal offsets produce different waveforms (example, with second waveform offset −0.45 ms relative to first, shown in (C). These summed waveforms lie along a trajectory in the feature space, parameterized by their temporal offset. Several examples (blue points) are shown in (D), along with their associated waveforms.

Clustering methods are generally successful when each neuron's spike waveform is sufficiently distinct from background noise and from those of other neurons, or when spikes occur primarily in isolation. However, these methods generally fail when spike waveforms from multiple neurons are superimposed [4], [10], [11], [30]. Specifically, if two neurons fire synchronously, the resulting voltage trace will resemble the sum of the individual waveforms [31]. The sum of the two spike waveforms forms a pattern that is distinct from the waveforms considered separately, and clustering methods will either assign the composite spike waveform to a distinct cluster–thus “hallucinating” a fictitious neuron–or discard the observation as an outlier that does not match any neuron. Figure 1 demonstrates the systematic failure to identify the near-synchronous spikes of two neurons recorded in primate retina [29], [32]. Figure 1A–B shows the superposition of synchronous spike waveforms, which a clustering method fails to identify. The problem is not limited to synchronous spikes, as shown in Fig. 1 C–D: any spikes whose waveforms exhibit non-zero dot product can give rise to an unrecognizable composite waveforms when superimposed. The feature-space trajectory of overlapping spikes can trace out regions of feature space distinct from the waveforms of each constituent neuron. These points will also typically be discarded as outliers by traditional clustering methods.

**Figure 2:**
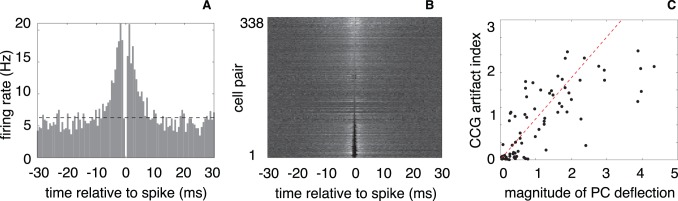
Cross-correlation artifacts induced by failure of clustering method for temporally overlapping spikes. (**A**) The cross-correlation function (CCF), which expresses the firing rate of one neuron relative to the spike times of another neuron. The CCF shows a substantial elevation in the firing of the primary cell in a time window extending roughly ± 5 ms around the spike of the secondary cell, as well as a sharp notch at the origin (width roughly ± 1 ms). The timescale of this notch matches the range of times over which the waveforms interfere with each other, as shown in Figure 1. (**B**) Summary of pairwise cross-correlations for all adjacent ON retinal ganglion cells within a simultaneously recorded population (338 pairs). Each row of the image represents the CCF between a pair of cells (shade of grey represents firing rate relative to the mean). Rows are sorted according to the value of the center time bin. (**C**) For pairs of neurons with significant synchronized firing, the magnitude of the secondary spike waveform (corresponding to the length of the red vector in Figure 1 ) provides a strong prediction of the strength of the CCF artifact (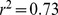). We quantify the strength of the CCF artifact (index on vertical axis) as the difference between the average firing rate during the intervals of 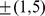 ms and 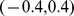 ms, divided by the baseline firing rate.

The failure to correctly identify near-synchronous spikes in a pair of neurons leads to an artifact that can be observed directly. Figure 2 A shows the cross-correlation function (CCF) between recovered spike trains of an adjacent pair of ON parasol retinal ganglion cells (RGCs), which are known to exhibit some synchrony in their spiking. The cross-correlation function provides an estimate of the instantaneous spike rate of the second cell relative to the time of a spike in the first cell. The plot in Figure 2 A shows an increase in rate over the interval 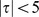 ms, which is typical for the timescale of synchrony in these cells [33]–[35]. But one can also see a pronounced CCF notch in the interval 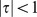 ms, which corresponds to the most highly synchronized spikes. This notch has been observed in extracellular but not intracellular recordings [34], [36], and its duration is matched to the interval over which the clustering failures identified in Figure 1 occur. These two facts suggest that the notch is an artifact that corresponds to spikes that the clustering method has failed to identify.

This sharp notch in the cross-correlogram is quite common. Figure 2 B shows a grayscale image whose rows are cross-correlograms between pairs of simultaneously recorded adjacent RGCs. The vertical black streak at 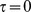 corresponds to the notch, and is seen to occur for many neuron pairs. Amongst synchronous cells, we can further demonstrate that the strength of the notch artifact is related to the geometry illustrated in Fig. 1. Intuitively, the magnitude of the waveform of the secondary cell determines how frequently spikes of the primary cell will fall outside of its cluster (and thus be classified as outliers). Figure 2 C quantifies this relationship, plotting a measure of the strength of the artifact against the magnitude of the secondary neuron waveform, across all pairs of adjacent RGCs. The significant correlation (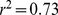) supports the interpretation that the notch is an artifact arising from failures of clustering for near-synchronous spikes.

### Estimating Spike Trains with Binary Pursuit

We formulate spike sorting as a statistical estimation problem. Specifically, we develop a generative model that describes how the measurements (extracellular voltage measurements) relate to the quantities to be estimated (spike times and spike waveforms). We also develop an algorithm for inferring spike times and waveforms from measurements under this model [21]. We provide a summary of our solution here; full details are provided in Methods.

Our model assumes that each neuron's spikes give rise to a characteristic space-time voltage pattern or “waveform” on the recording electrodes. Spike waveforms may extend several milliseconds in time and across many electrodes, depending on the three-dimensional layout of electrodes and neurons, as well as their electrical properties. We denote the waveform of the *j*’th neuron with a vector-valued quantity, 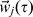, which has indices across all electrodes at each time 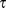 relative to a spike time. We assume the voltages measured across electrodes during an experiment are a sparse linear superposition of these spike waveforms, contaminated with background noise, 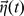:
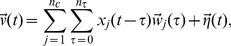
(1)where 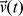 is a vector-valued function of time whose components contain the raw voltage traces recorded on each electrode, and 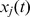 is a binary variable that indicates whether the *j*th neuron has spiked at time 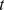. Note that we have discretized time (i.e., 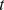 takes on integer values corresponding to discretized time bins), in anticipation of a numerical optimization algorithm that will be implemented on a digital computer. The sum over time represents a convolution of each waveform 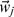 with the corresponding neuron's spike train 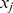. The constant 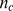 is the number of neurons in the population. The constant 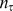 is the number of time steps in the spike waveform (assumed the same on all electrodes for all neurons).

We assume the probability distribution of the background noise can be approximated as a multivariate Gaussian, which specifies the conditional probabilistic relationship between the desired spike times and waveforms, and the observed electrode voltages:
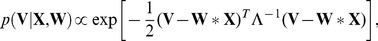
(2)Where **V**, **W**, and **X** are vectors containing the full content of 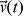, 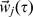, and 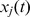 across space and time, and the bilinear term 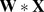 denotes the convolution expressed in Eq. 1. Note that 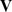 denotes the vector formed by taking the entire 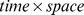 matrix of recorded electrode data and reshaping it into a single column vector, while 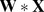 denotes a vector of the same size, formed after temporally convolving the waveform matrix 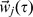 with the binary spike train 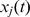 for each neuron and summing across neurons.

The covariance matrix 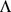 characterizes the spatiotemporal covariance of the noise in the recorded voltage signal, which is largely due to background electrical activity in the surrounding neural tissue(some of which may be due to spikes that are too small to reliably detect), and exhibits strong correlations in space and time, particularly for dense arrays. We discuss estimation of 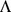 in Methods.

To complete the generative model, we need to specify prior probability distributions over the spike trains 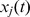 and spike waveforms 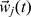. For spike trains represented at high temporal resolution, a natural prior is a Bernoulli distribution:
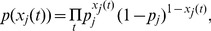
(3)where 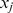 is a binary variable representing a spike (or lack thereof) for the 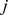
th neuron, in a single time bin 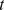. The parameter 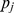 specifies the prior probability that a time bin contains a spike, and is generally quite small. Given a voltage sampling rate of 20,000 Hz, for example, a neuron spiking at 40 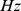 emits an average of one spike per 500 bins, corresponding to a Bernoulli prior with 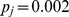. This prior assumes that spikes in different time bins, and for each neuron, occur independently. Finally, we imposed a sparseness penalty on the spike waveforms 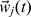, exploiting the fact that the waveforms tend to be localized across electrodes, and to reduce the computational cost of inference (see Methods for details).

This completes our generative model, consisting of a likelihood 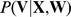 and priors 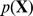 and 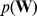. From these ingredients, we can use Bayes' rule to obtain the posterior distribution over spikes and waveforms given the data: 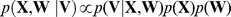. Our goal here is to develop a computational algorithm for maximizing this posterior, that is, to obtain the *maximum a posteriori* (MAP) estimate of the spikes and waveforms. (See [16] for a discussion of more general Bayesian inference methods, which can be made tractable for much lower-dimensional data). The negative log-posterior provides a quadratic objective function that we will seek to minimize for 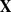 and 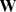:
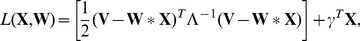
(4)where 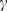 is a vector of constants that depend on the prior Bernoulli spike rates 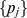. In essence, this objective function consists of two terms that impose differing constraints on the solution. The first is the squared error between the linear superposition of spike waveforms and the voltage data (measured in the space of the noise covariance). The second, which comes from the Bernoulli prior, places a penalty on each spike, and thus serves to reduce the number of spikes. The penalty (cost per spike) differs for each cell, and is derived from the prior probability of spiking in that cell (see Methods).

This is a hybrid discrete/continuous objective function (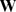 is continuous, 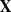 contains binary spikes), and there are no known methods for finding unique global minimum apart from brute-force search. Instead, we search for a local minimum using *coordinate descent*, which involves alternating between solving for each of these unknowns while holding the other fixed. Specifically, the algorithm uses the following steps:

1. Initialize using a standard clustering algorithm to identify the number of neurons and their approximate firing rates 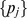.
2. Estimate the spike waveforms for all neurons across all electrodes by minimizing the objective function 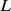 (Eq. 4 ) for 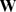; this is a simple least-squares linear regression problem.
3. Estimate the noise covariance 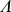 from the residual prediction errors, then whiten the data by the inverse square root of 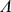 and re-estimate waveforms 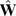.
4. Estimate spikes by minimizing 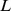 for 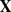. This is a sparse binary linear inverse problem [37], and the exact solution is intractable. Instead, we develop a greedy method that we call *binary pursuit*. Binary pursuit greedily inserts and removes spikes so as to maximally decrease the objective function until a local optimum is reached.
5. Return to step 1 and repeat until the estimated spike times and waveforms change minimally.

We provide the full details of this algorithm, along with practical and theoretical justification, in Methods.

### Performance Comparison

To evaluate our algorithm, we examined data recorded with a multi-electrode array in primate retina [29]. The custom 512-electrode array samples electrical activity at 20 kHz, providing approximately 30 samples for each 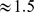
ms action potential (Fig. 1 a) [32]. This data set contains 364 identified retinal ganglion cells, spiking at an average rate of 10 sp/s. This dataset is especially challenging due to the high degree of multiplexing: each electrode records spikes from many different neurons, and each neuron projects to many (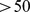) electrodes. Spike superposition is exacerbated by the fact that mammalian retina exhibits substantial synchronous spiking activity [33], [38], [39].

**Figure 3:**
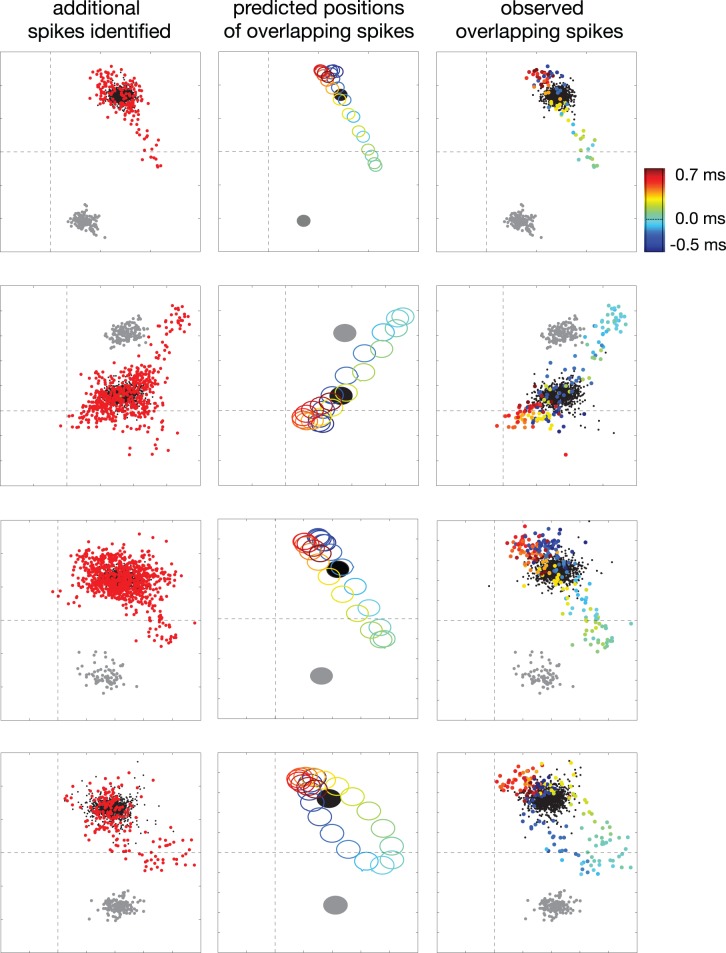
Comparison of spikes estimated using clustering and binary pursuit. Each row shows results for one example neuron. Each plot depicts the 2D linear feature space used for clustering (see Fig. 1 ). **Left column:** Black and gray points indicate spikes obtained by clustering for two cells. Additional spikes obtained for the black cell by binary pursuit (but ignored as outliers by clustering), are scattered in various directions relative to this ellipse (red points). Note that some points do not appear to be outliers within the two dimensions shown, but are outliers in other dimensions. **Middle column:** When the spikes of these two cells overlap in time, the resulting superimposed waveform is predicted to lie along a trajectory (see Fig. 1 ). Filled black and gray ellipses correspond to the location of isolated spikes for the primary and secondary cells, respectively. Size and shape of ellipses corresponds to the level curve (at one standard deviation) of the estimated (Gaussian) noise distribution. Colored ellipses indicate predicted locations of noisy superimposed waveforms, with color indicating their temporal offset. **Right column:** Subset of spikes identified by binary pursuit that were either isolated (black and gray points), or overlapping (colored points, with color indicating the temporal offset of the two spikes).

We compared spike train estimates obtained with traditional clustering and with binary pursuit. The most immediate difference was that binary pursuit identified a larger number of spikes for every cell. These additional identified spikes generally overlapped the spikes of other cells, as illustrated in Fig. 3. The left column shows the spikes of four example cells obtained using a clustering method.

For each example cell, the spikes of a second cell recorded on similar electrodes are also shown (gray points). Binary pursuit identifies a number of additional spikes, which are scattered in multiple directions away from those identified by clustering (red points). The red points are incorrectly classified as outliers by clustering. (Note that some points do not appear to be outliers within the two dimensions displayed, but are outliers along other dimensions.) The middle column shows the predicted locations of the superpositions of the spike waveforms of the two cells with different temporal offsets. The right column shows additional spikes of the primary cell identified by binary pursuit, color coded according to whether they overlapped a spike of the second cell, and if so, at what temporal offset. The estimate spike times are consistent with the predicted superpositions in the middle panel. Note that synchronous spikes (zero temporal offset) deviate furthest from the cloud of isolated spikes.

**Figure 4:**
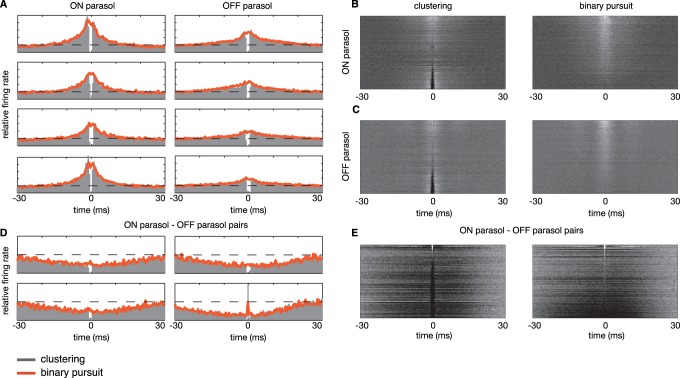
Cross-correlation artifacts introduced by clustering techniques are greatly reduced with binary pursuit. (**A**) Cross-correlation between four distinct pairs of adjacent ON parasol cells (left column) and OFF parasol cells (right column), for spike trains estimated using clustering (gray bars) and binary pursuit (red line). Dashed line indicates baseline firing rate. (**B–C**) Summary of cross-correlations between adjacent pairs of neurons (338 ON and 369 OFF neuron pairs), with spike trains obtained from clustering (left column) and binary pursuit (right column). Within a single image, each row represents the cross-correlogram between a single pair of neurons, with intensity indicating firing rate relative to mean rate. Rows are sorted by the firing rate of the bin at 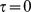. The artifactual notch at zero that arises from cluster-based sorting is now visible as a dark streak at 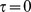, and largely disappears with binary pursuit sorting. (**D**) Cross-correlation between four distinct pairs of adjacent ON and OFF parasol cells. (**E**) Summary of cross-correlations between 225 pairs of adjacent ON and OFF parasol cells.

We also compared the cross-correlations of spike trains estimated with binary pursuit and clustering. Figure 4 A shows examples for eight pairs of adjacent parasol cells (four ON, and four OFF pairs). As shown in Fig. 2, the clustering method leads to an artifact in the CCF (a notch at 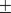 1 ms), but this artifact is reduced or eliminated for the spike trains estimated using binary pursuit. Figure 4 B summarizes this improvement across all pairs of ON and OFF parasol cell in a single recording. Cells of opposite polarity are known to exhibit weak anti-correlation [33], [40], as can be seen in cross-correlations of four example ON-OFF pairs, shown in Fig. 4 C. Again, the clustering method produces an artificial notch at the origin, indicating a failure to correctly identify spikes that are near-synchronous, and this artifact is systematically removed under binary pursuit. A summary across the population is shown in Fig. 4 D. Curiously, on a small fraction of cell pairs, a spurious peak in cross-correlation is observed even for binary pursuit, which we believe reflects a lack of discriminability of the two waveforms (see Discussion).

**Figure 5:**
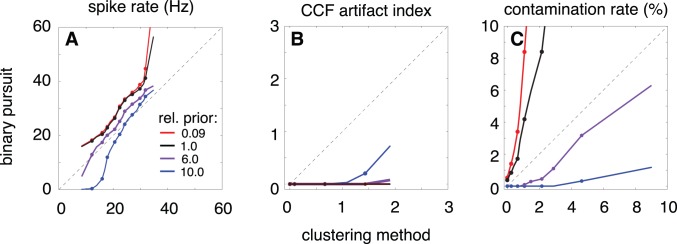
Comparison of spike trains estimated using binary pursuit and clustering. Three different summary statistics are computed and compared for 293 retinal ganglion cells. For each statistic, the data are shown as “Q–Q” plots: Each line spans the range of quantiles from 5% to 95%, and points are plotted at corresponding deciles of the distributions from 10% to 90%. Different colored lines correspond to different Bernoulli spike rate priors: values in legend indicate a multiplicative factor on the log-prior, relative to the firing rate estimated from clustering. (**A**) Spike rate. (**B**) Cross-correlation function artifact index measures the depth of the “notch” at the origin of the cross-correlation function between a pair of cells, a measure of missed spikes. (**C**) Refractory period contamination rate, which is a measure of false positives. Note that the purple curves (which arise from using a prior for each cell that is six times the firing rate of spikes estimated using clustering) show a reduction in both contamination and CCF artifacts relative to clustering.

The black curves in the panels of Fig. 5 summarize the relative behavior of the two spike sorting methods. Figure 5 A shows that binary pursuit identifies more spikes for every cell in our population (N = 293 cells). Figure 5 B shows a comparison of the magnitude of the CCF artifact. The spike trains obtained using binary pursuit are seen to have little or no artifact. From these two plots, one might be tempted to believe that binary pursuit has solved the spike sorting problem. But further examination reveals a new problem: an increase in refractory-period violations, which provide another indicator of spike-sorting errors [4], [15], [24], [41]–[43]. We quantify these errors in terms of the “contamination rate” for each neuron, defined as the ratio of the frequency of occurrence of spikes within the refractory period (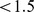 ms) to the baseline frequency of spikes outside this window. (A contamination rate of 50% indicates that the rate of spikes detected during the refractory window is equal to half the rate of spikes detected outside this window). Figure 5 C shows a comparison of the contamination rate for spikes sorted by clustering and binary pursuit. We see that for a large proportion of the cells, binary pursuit has a significantly higher contamination rate than clustering, and thus some of the increase in spike rate seen in these cells is likely due to inclusion of erroneous spikes.

Spike sorting is a type of signal detection problem, and it is well known that failures in such problems come in two forms: *misses* (in which a true spike is not detected), and *false positives* (in which an artificial spike is inserted). The CCF artifact provides a measurable indicator of misses, whereas the contamination rate is a measurable indicator of false positives. In classical signal detection theory, misses and false positive errors trade off against each other as one adjusts the decision threshold [44]. In the context of a Bayesian approach, one may accomplish this tradeoff by adjusting the prior probability on signal occurrence. This idea may be used directly with our spike sorter to trade off the CCF artifact against the contamination rate, as shown in Fig. 5. Reducing the Bernoulli spike rate decreases the number of estimated spikes, increases the CCF artifact, and decreases the contamination rate (Fig. 5 A–C, blue curves). However, a more moderate reduction in the Bernoulli rate results in a contamination rate significantly below that of clustering, while minimally increasing the CCF artifact index (Fig. 5 B–C, purple curves). Thus, for these data, there exist prior settings for which both types of errors occur less frequently than with clustering.

### Estimating Error Rates in the Absence of Ground Truth

The results of Fig. 5 show that spikes sorted with binary pursuit depend significantly on the choice of prior spike rate, and suggest that this value could be selected to simultaneously minimize both the CCF artifacts (misses) and the refractory contamination (false positives). These two measurable errors are only *proxies* for the true errors that one would like to minimize. In general, one does not know the true errors and we cannot assume that the true errors are proportional to their corresponding measurable quantities.

**Figure 6:**
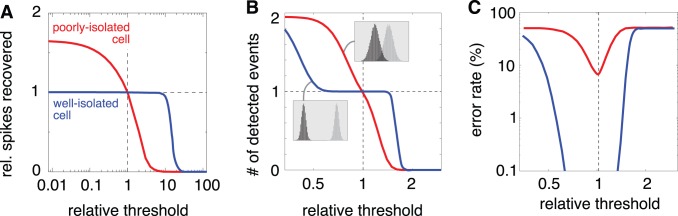
Sensitivity of number of spikes recovered to the prior on spike rate. (**A**) Results for two example cells, one well-isolated (blue), and one poorly isolated (red). Adjusting the Bernoulli prior parameter (for each cell individually) alters the threshold used for spike identification (see Methods), which leads to an increase or decrease in the number of estimated spikes. (**B**) Simulation of detection of a scalar signal contaminated by Gaussian noise, for two different SNRs. Insets indicate histograms of noise observations (black) and signal observations (gray). The number of detections (“hits” plus “false positives”) varies with the choice of threshold, and the shape of the curve depends on the SNR. (**C**) Error rates (“misses” plus “false positives”) as a function of threshold for the simulations in (B).

We can use signal detection theory to develop a method for assessing the error rate of individual neurons in the absence of ground truth. This can be used both to select prior values for each cell, and to determine which neurons have acceptable spike sorting errors. The method is based on a simple observation: In a Bayesian setting, if an estimate is well constrained by the data, then the value of the prior parameter has little effect [45]. Thus, if the spike waveform of a cell is easily distinguished from the background noise and from the waveforms (or superpositions of waveforms) of other cells, the number of spikes found for that cell should be insensitive to the parameter value chosen. Fig. 6A illustrates this effect by showing the sensitivity of spike count to the Bernoulli prior parameter for two different RGCs. The well-isolated cell shows a spike count that is stable with respect to changes in threshold up to an order of magnitude in either direction. In contrast, the poorly-isolated cell is highly sensitive to the threshold value.

This behavior is nearly identical to that obtained from simulation of a simple signal detection problem. Figure 6 B shows results for detecting a scalar value from a scalar measurement corrupted by additive Gaussian noise. Optimal detection (in the sense of minimizing errors) is achieved by thresholding the measurement at a value that depends on the prior probability of occurrence of the signal [46]. For high SNR, the number of detected events is stable over a broad range of thresholds, whereas for low SNR, the number of detected events is highly sensitive to the choice of threshold. This sensitivity provides an indication of how cleanly the signal can be isolated from the noise, which is directly related to the error rate in the two situations, as illustrated in Fig. 6 C.

**Figure 7:**
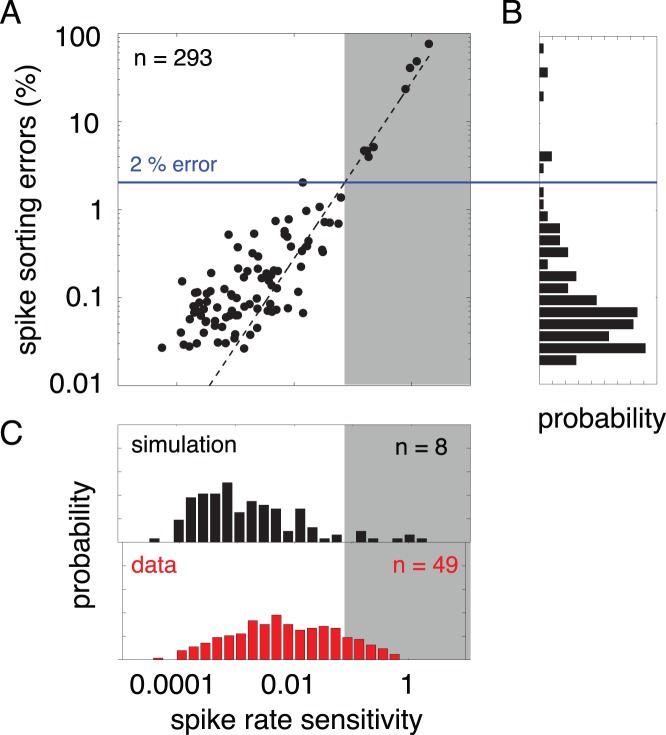
Quantifying the robustness of spike sorting in the absence of ground truth through a prior sensitivity analysis for all parasol cells (). 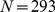
 (A) The sensitivity of the spike rate to the prior distribution plotted against the spike sorting error rate in simulated data (see text for details). Note that both axes are plotted in logarithmic space. Dashed line is best fit line (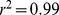). Gray box indicates spike rate sensitivities achieving 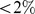 error. (B) Distribution of error rates across simulation. The solid blue line indicates 2% error rate in simulation. (C) The distribution of spike rate sensitivities from simulation (top) indicate that 8 cells contain spike rate sensitivities which imply an 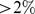 error rate. Distribution of spike rate sensitivities calculated from real data suggest that 49 cells contain 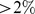 error rates.

To make use of this relationship in spike sorting, we need to estimate the relationship between the sensitivity and the error rate. We simulated 120 seconds of electrode data using the generative model of Eq. 1 for 293 neurons, and estimated the spikes of each neuron using binary pursuit. We recomputed these estimates while varying the prior of each neuron individually. Figure 7 A shows a scatter plot of the relationship between the sensitivity (quantified as the derivative of the spike count with respect to the threshold for each neuron), and the spike sorting error rate in the simulated data. The data are reasonably well fit by a power law (straight line fit on a log-log plot, 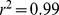).

As an example of the use of this relationship, suppose one wanted to analyze only those neurons with a spike sorting error rate less than 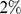. Using the scatter plot of Fig. 7 A, we find that that the estimated spike trains for 285 of the simulated neurons had error rates 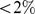 (Fig. 7 B). We then use the relationship between sensitivity and error rate to estimate the error rates in the real data. Figure 7 C suggests that the 49 neurons with a spike rate sensitivity 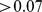 are likely to have error rates 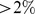.

## Discussion

We have formulated the spike-sorting problem in a statistical estimation framework based on a generative model of extracellular electrode data. The model, while extremely simple, provides an explicit statement of the assumptions underlying our methodology: the recorded voltage traces arise as a linear superposition of spike waveforms from multiple neurons, along with additive correlated Gaussian noise, with a prior on the frequency of each neuron's spikes.

We have shown that clustering methods, which are the current *de facto* standard for sorting spikes, exhibit systematic failures, arising from an implicit assumption that the spike waveforms contained in the recorded voltage traces do not overlap. We developed binary pursuit, an algorithm for finding a (local) maximum of the posterior expressed by our model, and demonstrated its capabilities in sorting multi-electrode data from the retina, using refractory violations and cross-correlation artifacts as measurable indicators of errors. In addition, we've shown that a statistical formulation of the estimation problem allows us to test the robustness of the spike sorting results to perturbations in the prior parameters, providing a measure of the quality of the results in the absence of ground truth.

### Relationship to Previous Work

Previous literature on spike sorting is quite extensive, but focuses mostly on variants of matched filtering or clustering [13]. The artifacts that can arise in these methods have been previously documented [10], [11], [13], [47], and a few authors have developed post-processing algorithms for repairing them [4], [11], [26], [28], [48]. Such repairs can be effective in some situations, but since they are generally not tied to any particular generative model, it can be difficult to state the conditions under which they will succeed. Several methods operate by identifying portions of the voltage trace that are likely to contain spikes, and then searching exhaustively for the combination of spikes (and temporal offsets) that can best explain them. This type of method can be quite effective for small numbers of cells, but the computational cost scales exponentially with the number of cells, rendering it intractable for large multi-electrode arrays.

One method closely related to our own uses a convex relaxation of the discrete (binary) optimization problem [25]. Specifically, the authors use an 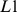-norm (or “lasso”) penalty on positive, real-valued spike coefficients [49]. The resulting objective function is identical to ours, but is convex on the augmented space of positive (as opposed to binary) coefficients, meaning that a unique global maximum can be obtained via quadratic programming. Spikes are then obtained by thresholding these coefficients. We have experimented with this approach on smaller datasets (using spike trains from 27 neurons on 76 electrodes, published in [50]). We found that the algorithm gave results of comparable quality to binary pursuit, but required an order of magnitude more computation time, making it impractical for datasets of the size considered here.

Recent work from Prentice et al [26] describes a method for Bayesian (MAP) spike train estimation that also has a number of similarities to our own. In fact, that paper provides a more complete method for spike-sorting, as it uses a clever method for clustering multi-electrode data and estimating the number of neurons (whereas we have relied a standard clustering method to initialize our algorithm). However, [26] does not specifically discuss cross-correlation artifacts or methods for assessing performance in the absence of ground truth. The dataset examined in [26] had a 30-electrode recording from 107 neurons with 1.5 sp/s average spike rate; this differs substantially from our dataset, which had a 512-electrode recording from 298 neurons with 10 sp/s average spike rate. Cross-correlation artifacts were likely a larger problem in our dataset due to the higher spike rates and higher density of neurons. Differences in the two algorithms reflect some of the differences in datasets. For example, [26] used periods of silence to estimate the noise covariance, and extracted isolated (multi-neuron) firing events from the raw datastream before sorting. By contrast, our recordings rarely exhibited total silence across the array, and single-neuron waveforms often extended across more than 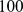 electrodes. This high degree of temporal and spatial overlap precluded the extraction of isolated “spiking event” data vectors, and required temporally traversing the entire raw datastream to estimate spikes. In this sense, our algorithm more closely resembles the methods of [4], [28], which also involve greedy subtraction of spikes from the raw data.

Taken together, it is clear that the recent literature has seen the development of several closely-related methods, all involving MAP inference under a generative model with Gaussian noise and a sparse prior on spike trains. We believe there is much to be gained by comparing, synthesizing and extending these algorithms to improve speed, computational complexity, accuracy, and robustness.

### Sources of Error

We cannot provide guarantees on the absolute performance of our algorithm, since performance is inherently limited by noise level, the number of neurons, and the discriminability of their waveforms. The presence of noise generally implies the possibility of errors, and one should think in terms of understanding and bounding the errors. In this regard, statistical formulation allows us to partition errors into three categories, and to separately consider improvements that might reduce each.

The first of these are irreducible model errors; that is, errors that would be incurred if the data actually arose from the process assumed in our model. One asks “what is the probability that any particular spike or combination of spikes might be mistaken for background noise, a different spike or combination of spikes.” This is a multi-dimensional signal detection problem, and the error rate will be a function of the amplitude and similarity of the spike waveforms (at all relative temporal offsets), relative to the amplitude of the noise. These errors can be examined through simulations (i.e., by applying the spike sorter to artificial data generated by drawing samples from the model), although it is important to recognize that such simulations will also include the effects of algorithmic errors (see next paragraph). Some authors have examined such errors (specifically, the tradeoff between hits and false alarms) in the context of single neuron spikes [51].

The second type of error arises from failures of the optimization algorithm. Our algorithm operates by taking iterative steps, each of which decreases the negative log-posterior; we can therefore guarantee that it will reach a local minimum. However, since the objective function is not convex, this minimum is not guaranteed to be a global minimum. The solution is also susceptible to numerical approximation errors (e.g., Taylor series), although careful implementation can ensure that these are not significant. The sparse linear inverse problem has become a focus of intense study over the past ten years, and the literature can be loosely partitioned into two general classes: the greedy pursuit methods (including iterative thresholding), and the convex relaxation methods (e.g., basis pursuit). The greedy methods (such as the one we have presented here) tend to make mistakes in which overlapping spike waveforms are “explained” with an incorrectly placed spike or combination of spikes. This could potentially be improved with post-processing, in which one examines those spikes or combinations of spikes that are most likely to generate superposition errors (e.g., [48]). We have also begun to examine relaxation methods [52].

The third type of error arises from incorrectness of the model. The most common of these are likely to be errors in the assumed waveforms or noise description. For example:

- the set of model waveforms might include a false waveform. For example, if a clustering method is used to obtain initial waveform estimates, two cells with a high degree of synchrony can result in identification of a false neuron associated with the combined waveform.
- the waveforms of real cells are variable, exhibiting slow drift or systematic changes in amplitude or shape (e.g., during spike bursts) [53], [54].
- the electrode “noise” does not arise from a Gaussian process, but primarily from the superposition of spikes of unsorted cells [9]. Although it is generally intractable to fully incorporate this into the model, some authors have modeled the non-Gaussianity of these signals using heavy-tailed noise distributions [55], [56].

We have deliberately designed our spike train model to be simple, but the basic framework can be extended to incorporate additional constraints on spike trains (e.g., refractoriness, joint activity, stimulus dependencies) or variability (e.g., priors on the waveform shapes, or on their drift in shape over time, [54]). In general, additional constraints serve to further restrict the set of possible solutions, which can improve the results if the constraints correspond to true properties of the neurons, and assuming they can be readily incorporated into the optimization algorithm. On the other hand, over-constraining the solution can lead to additional “Miss” errors. Similarly, the model could be relaxed to allow more substantial variability in the spike waveforms, but if this enlarges the set of possible solutions and can thus open the door for additional “False Positive” errors.

### Future Directions

We have focused on the problem of identifying spikes under the assumption that the number of neurons is known. (Specifically, we used a clustering analysis to estimate the number of neurons). A full solution to the spike sorting problem should incorporate uncertainty about the number of neurons as well. Recently developed non-parametric Bayesian clustering methods based on the Dirichlet process, which do not yet take account of superposition but might be extended to do so, provide one promising direction for future work [16]. Another important direction is to improve the speed and computational efficiency of our method, either through parallelization or perhaps through greedy methods that employ binary pursuit only in restricted spatio-temporal regions of the recording (i.e., where a region of spike overlap can be identified through an increase in residual error). Further improvements might be achieved by explicitly modeling temporal dependencies in spike trains [43], [53], tuning information [58], [59], non-stationarity of spike waveforms (due to shifts in tissue or biophysical changes in the neurons themselves [53], [57]), and non-stationarities in the noise distribution. In our view, the primary virtue of a model-based approach is that it requires formalizing one's assumptions about the statistical structure of the data, making it possible to achieve improvements either by identifying and replacing inaccurate assumptions, or by observing new statistical features of the data that can make the problem easier.

### Summary

We have provided a thorough analysis of superposition errors that arise in clustering-based methods, a new spike-sorting algorithm based on a generative model that allows for spike overlap, and accompanying methods for assessing the robustness of the estimated spike trains. These results provide a principled and self-consistent formulation of the problem that can serve as a substrate for the development of new model-based spike sorting methods.

## Methods

### Mathematical Details of Sorting Algorithm

Our algorithm seeks to maximize the joint posterior 
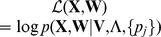
 (Eq. 4 ) over spike trains 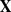 and spike waveforms 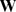 given the voltage data 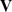, the noise covariance 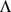, and prior spike probabilities 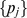. Our general inference strategy is to maximize the log-posterior via coordinate ascent, which means alternating between maximizing 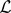 for 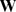 and for 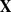. This procedure is guaranteed to converge to a local maximum of the posterior.

The geometry of the log-posterior informs our optimization strategy, and may in the future be exploited to design improved spike train estimators. The expected voltage is a bilinear function of 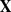 and 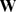. Gaussian noise implies that maximizing 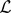 for 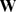 given 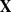 is a linear least squares problem, which can be solved efficiently by linear regression. Maximizing 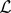 for 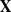 given 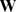 is also a linear least squares problem, due to the fact that the log Bernoulli prior (Eq. 3 ) is linear in 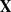. However, the discreteness of 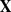
–each component must be zero or one–means that this optimization is a non-convex problem. We therefore resorted to a greedy algorithm for estimating 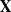 given 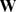. However, the convex relaxation that results from allowing scalar-valued 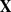 in the interval 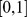 *does* does have a unique global maximum. Spike sorting methods that make use of this scalar solution for initializing a search over binary spike trains may provide one promising avenue for future research (see [25]). We implemented this method but did not find any substantial improvement over the current algorithm, suggesting that the additional computational cost of such an approach is not justified for the recordings considered here. We summarize the details of our algorithm below.

### Waveform Estimation

We begin by estimating the spike waveforms 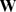 using an initial estimate of the spike trains 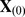, the latter of which is provided by a clustering-based method (see Methods). The rationale for estimating 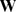 first is that the clustering-based method uses a low-dimensional linear feature space derived from a small neighborhood of nearby electrodes (depicted in Figs 1 and 3), and we would like to learn each neuron's full spatiotemporal spike waveform across all electrodes to better identify spikes.

Given 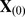, we maximize the posterior (Eq. 4 ) for 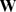 using an initial assumption of independent noise (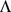 equal to the identity matrix). This yields the solution:
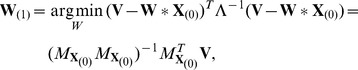
(5)where 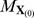 is a toeplitz matrix formed from the elements of 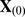 such that 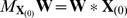. This solution minimizes the quadratic term in 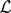.

We then *prune* 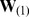 by subset selection [60] on the vector norm of 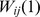, the waveform of the 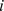
’th neuron on the 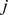
’th electrode. That is, we set 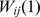 to zero if 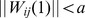, where *a* was a constant multiple of the noise on the 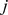
’th electrode. Subset selection effectively induces sparsity on the estimate of 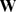 (see, e.g., [49], [50], [61]–[63]), which regularizes and reduces computational cost, but does not bias estimates of large-amplitude waveforms.

### Noise Covariance and Whitening

The next step is to estimate the noise covariance 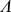 from initial estimates of the spike trains 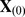 and waveforms 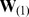. Knowledge of this covariance will allow us to *sphere* the noise so that it is independent in time and across electrodes [64]. This will transform the first term in the log-posterior (Eq. 4 ) from a weighted to an unweighted sum of squares, which reduces the computational cost of spike train estimation.

We could in principle estimate 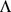 using the covariance of the residual errors in predicting 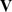, that is, 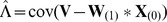. However, this matrix is far too large to estimate, or even to store in memory. We therefore modeled the noise as having a separable space-time correlation structure, with a limited extent in time. This allowed us to whiten the data using a step-wise whitening procedure: first, we estimated the temporal noise covariance 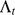 on each electrode using a 16 time-bin (0.8 ms) window, and then filtered the data from that electrode with the central column vector of 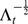. Then, we estimated the instantaneous noise covariance 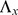 across all 512 electrodes in the array (a 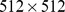 matrix) and multiplied the vector of data in each time bin by whitening matrix 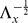.

Let 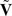 denote the whitened electrode data obtained from this two-stage whitening procedure. (The residuals of 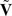 had approximately flat autocorrelation in both time ans space, indicating that the assumption of space-time separable noise was a reasonable assumption). We then re-estimated and sparsified the waveforms (as described above) to obtain 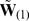, the whitened spike waveforms.

### Spike Train Estimation

The most computationally intensive step in the algorithm is estimating the set of spike trains 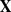 given 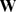. This involves maximizing the log-posterior in the space of whitened voltage signals, which can be written:
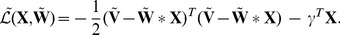
(6)


The final term 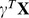 arises from the Bernoulli prior over each neuron's spike train. We initialize the prior probability of a spike in each neuron using 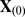, the spike train estimate returned by clustering-based method (see Methods). We set 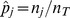, where 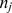 is the number of spikes from the *j*’th neuron, and 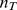 is the total number of time bins in the experiment. The weights 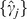 composing 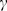 are then given by
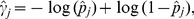
(7)which follows from the fact that the log of the prior (Eq. 3 ) can be written 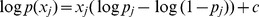.

As noted above, maximizing 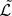 for 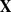 is a quadratic optimization problem on a binary lattice, since each element of 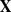 is 0 or 1. The advantage of working in the whitened space is that the log-posterior is just the sum of the residual errors plus the penalty term from the Bernoulli prior; when inserting or a removing a particular spike, we need only compute the change in residuals on the bins where the expected voltage 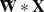 changes, i.e., the electrodes and time bins affected by a particular spike waveform.

Greedy binary optimization procedes as follows. Let 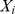 denote the 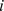
th bin of 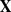 and let 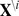 denote the vector 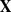 with the 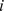
th bin removed. Let 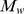 denote the (highly sparse) toeplitz matrix for convolution of waveforms with the spike trains, so 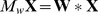. Let 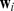 denote the *i*th column of the waveform matrix 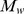, and 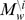 denote the same matrix with the *i*th column removed. We can now evaluate 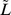 with 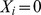 and 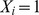 in order to determine whether the bin should contain a spike or not. Assuming that noise variance 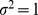 after whitening, we have, for all *i*:
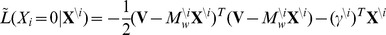


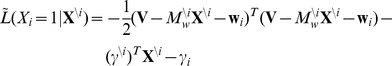
(8)


The difference gives the change in the log-posterior for changing 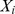 from 0 to 1:
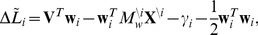
(9)and 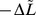 gives the change in the log-posterior for changing 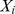 from 1 to 0. We can compute this difference for every bin 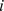, with initial setting 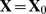. An obvious strategy for maximizing the posterior is then to proceed greedily, selecting the bin *i* for which 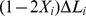 is largest, and flipping 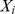 from 0 to 1 or vice versa, as determined by the sign of 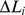. This strategy leads to a highly efficient computational algorithm, since after flipping a bin 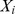, we only need to update 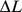 in the bins *j* for which 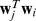 is non-zero (i.e., only bins nearby in time and space to neuron *i*). Moreover, we can pre-compute 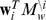 for all *i*, making it extremely fast to perform updates to 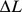 (eq. 9) following a spike insertion or deletion.

To reduce the computational cost of searching for the maximum of 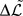, we processed the data in 1 s blocks. This made 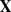 a vector of length 512×20,0000 = 10,240,000 for each block. The algorithm terminates when 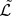 can no longer be increased by inserting or deleting a spike in any neuron in any time bin.

The full MAP inference algorithm (summarized in *Algorithm 1* below) involves coordinate ascent, which involves cycling through and re-estimating 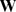, 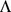, and 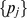 in turn as described above, repeating until the log-posterior cannot be increased further. In practice, however, the high cost of running multiple rounds of coordinate ascent, and the relatively good performance achieved with a single round of updates led us to stop with 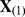, the spikes obtained from the first maximization of 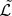 for 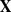.

**Algorithm 1: MAP inference procedure**

1. Estimate waveforms **W** by linear regression given voltage data **V** and initial spike train estimate 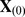.
2. Prune 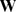, removing unnecessary electrodes from each neuron's spike waveform via subset selection (or other feature selection method).
3. Compute the residuals 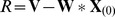 and estimate noise covariance 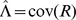.
4. Whiten by the square root of the inverse covariance: 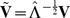 and 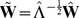

5. Estimate prior spike probabilities for each neuron: 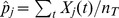

6. Estimate spike trains 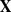 via binary pursuit given 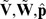.
7. Return to 1; Repeat until convergence.

Empirically, we found that sorting with the sparsity penalty 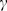 determined from the “plugin” estimate for the Bernoulli parameter 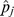 (Eq. 7) led to an undesirably large increase in contamination rate for many cells (see Fig. 5). For this reason, we systematically varied 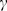 by a multiplicative factor, and found that a reasonable tradeoff between CCF artifact and contamination rate was obtained with penalty increased by a factor of 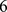, giving 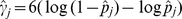.

### Accounting for Spike Waveform Variability

The algorithm described above assumes that spikes occur on a fixed lattice of discrete time points (with 0.05 ms spacing, given the 20 KHz sampling of our data). One consequence of this discretization is that “true” spike waveforms present in the analog voltage trace may be shifted relative to the waveform templates subtracted or added during binary pursuit. To address this form of aliasing error, we used a local expansion of the waveform of each neuron to account for shifts in the exact spike time and variations in the spike amplitude and spike width. This additional flexibility allows us to resolve spike times to a finer resolution than the sampling rate of the analog trace, and to account for variability in spike waveform height and amplitude that arises (for example) during bursting activity.

We account for such variability by assuming that a spike waveform 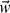 can vary slightly in time 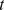 (relative to the discrete time lattice), amplitude 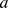 or width 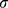 each time it appears in the data. Specifically, we represent each spike in the data using a local Taylor series approximation centered on the “canonical” waveform:
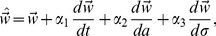
(10)where the waveform derivatives can be computed numerically:



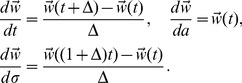
(11)For the derivative with respect to spike width 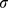, we interpolate the waveform and center it so that 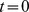 corresponds to the waveform peak; this ensures that the time-dilation 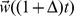 increases the width without shifting peak location. The basic intuition here is that, for smooth waveforms 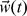, small shifts in spike time, amplitude, or width can be closely approximated by adding a small amount of the appropriate waveform derivative. (See [52] for a more direct embedding of this idea in a convex relaxation scheme known as *continuous basis pursuit*.).

For each observed spike in the dataset, the weights 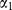, 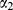 and 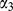 must be estimated in order to determine the exact spike time, amplitude and width. We simplify the formula above by expressing the “corrected” waveform 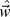 in matrix notation.
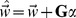
where 
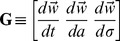
 and 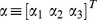


If we assume that a single spike occurs, then we can express the unknown 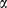 in terms of the voltage signal.
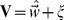


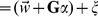
(12)where 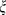 is a zero-mean Gaussian noise. Given this, the least-squares value of 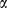 may be obtained as: 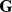:



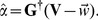
(13)The pseudo-inverse 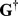 can produce large values of 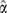 when the data exhibits large deviations from the true waveform, causing unrealistically large changes in spike width or amplitude. We can keep the correction small by adding an L2 (“ridge”) penalty 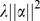, which shrinks 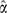 toward zero and results in the formula:
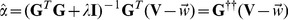
(14)


We set the regularization parameter 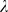 to minimize contamination errors in cross-validation data. Note that the new matrix 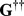 can be pre-computed for each waveform 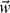 and applied to any residual 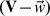 before maximizing the log-posterior to solve for the spike times. We incorporated this update rule into the binary pursuit algorithm described above, using it to update the residual error between 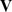 and 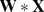 whenever a spike was added to 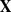.

In our dataset, we found that the temporal derivative term made the largest contribution to performance, and that the resulting estimates exhibited far fewer “doublets”, where the algorithm erroneously inserts two spikes from the same neuron in adjacent time bins.

### Clustering Method

In our multi-electrode recordings from primate retina, each electrode samples electrical activity at 20 kHz, providing approximately 30 samples for each 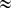
1.5 ms action potential (Fig. 1 a), and each spike typically elicits voltage signals occur across multiple electrodes, reflecting electrical propagation through dendrites, soma and axon (Fig. 1 b; see also [65]).

To obtain initial estimates of the spike waveforms present in a recording, we use a standard clustering methodology. The basic steps can be summarized as follows:

1. For each “center” electrode, identify candidate spikes via thresholding, and create a vector of the voltage data from a 1.5 ms window of time and neighborhood of 6 immediately neighboring electrodes.
2. Reduce dimensionality of the resulting collection of vectors using PCA.
3. Cluster the resulting vectors and identify the points in each cluster as the spikes of a single neuron, with human oversight to determine the number of clusters and assess the reliability of cluster assignment.

The spike sorting literature contains an extensive treatment of such methods [13], [20], [21].
